# Volatile Organic Compounds from Cassava Plants Confer Resistance to the Whitefly *Aleurothrixus aepim* (Goeldi, 1886)

**DOI:** 10.3390/insects14090762

**Published:** 2023-09-13

**Authors:** Thyago Fernando Lisboa Ribeiro, Demetrios José de Albuquerque Oliveira, João Gomes da Costa, Miguel Angel Martinez Gutierrez, Eder Jorge de Oliveira, Karlos Antonio Lisboa Ribeiro Junior, Henrique Fonseca Goulart, Alessandro Riffel, Antonio Euzebio Goulart Santana

**Affiliations:** 1 Institute of Chemistry and Biotechnology, Federal University of Alagoas, Maceió 57072-900, AL, Brazil; 2 Embrapa Food and Territories, Maceió 57020-050, AL, Brazil; 3 Natural Product Research Laboratory (LPqRN), Campus of Engineering and Agrarian Science, Federal University of Alagoas (UFAL), Maceió 57072-900, AL, Brazil; 4 Embrapa Mandioca e Fruticultura, Cruz das Almas 44380-000, BA, Brazil

**Keywords:** *Manihot esculenta* Crantz, Euphorbiaceae, repellence activity, terpenes, semiochemicals

## Abstract

**Simple Summary:**

Cassava (*Manihot esculenta* Crantz; Euphorbiaceae) plants have a long lifecycle. They remain in the field for at least 10 months and are exposed to numerous pests. Seventeen groups of pests that affect this crop have been identified. These include 35 species found in the Americas, 11 in Africa, and 6 in Asia, totalling approximately 200 arthropod species that feed on cassava. Whiteflies are considered one of the main pests of cassava worldwide. In northeastern Brazil, the most common whitefly species causing severe damage to cassava is *Aleurothrixus aepim* (Goeldi, 1886) (Hemiptera: Aleyrodidae). Cassava plants emit volatile organic compounds and secrete extrafloral nectar; these substances may be essential for developing biological control strategies against pests. Herein, we describe variations in the blend constitution and individual concentrations of constitutive volatile organic compounds released by two cassava genotypes. Additionally, we highlight the activity of a monoterpene, considering that the resistant genotype emitted higher concentrations of this compound.

**Abstract:**

Cassava is an essential tuber crop used to produce food, feed, and beverages. Whitefly pests, including *Aleurothrixus aepim* (Goeldi, 1886) (Hemiptera: Aleyrodidae), significantly affect cassava-based agroecosystems. Plant odours have been described as potential pest management tools, and the cassava clone M Ecuador 72 has been used by breeders as an essential source of resistance. In this study, we analysed and compared the volatile compounds released by this resistant clone and a susceptible genotype, BRS Jari. Constitutive odours were collected from young plants and analysed using gas chromatography–mass spectrometry combined with chemometric tools. The resistant genotype released numerous compounds with previously described biological activity and substantial amounts of the monoterpene (*E*)-β-ocimene. Whiteflies showed non-preferential behaviour when exposed to volatiles from the resistant genotype but not the susceptible genotype. Furthermore, pure ocimene caused non-preferential behaviour in whiteflies, indicating a role for this compound in repellence. This report provides an example of the intraspecific variation in odour emissions from cassava plants alongside information on odorants that repel whiteflies; these data can be used to devise whitefly management strategies. A better understanding of the genetic variability in cassava odour constituents and emissions under field conditions may accelerate the development of more resistant cassava varieties.

## 1. Introduction

Cassava (*Manihot esculenta* Crantz; Euphorbiaceae) is a substantial source of carbohydrates in Latin America and Africa [1]. Despite being stigmatised as a ‘third-world crop’, cassava has been the focus of research in critical areas such as better cultivars, resistance to pests and diseases, and better-quality seeds, considering its importance as a food crop and potential as a commodity in the broader economy [2,3,4,5]. Cassava is a subsistence crop. However, it is rapidly evolving as a cash crop and raw material for the production of starch, energy, and livestock feed in countries that are its major producers, such as Nigeria, Thailand, Indonesia, and Brazil. It can also be used as a raw material in the baking, food, and pharmaceutical industries [6]. Cassava is sensitive to low temperatures and is predominantly cultivated in tropical and subtropical regions; it has been grown by millions of small farmers in more than 100 countries [7]. This crop is well adapted to the climate and soil conditions of these countries. For example, its cultivation is widespread throughout Brazil [8], the fourth largest producer worldwide, with 18 million tons of cassava root being produced annually over an area of 1.24 million hectares [8]. Cassava is grown in different regions and has a long lifecycle; it remains in the field for at least 10 months and is exposed to many pests. Seventeen groups of pests that affect this crop have been identified. These include 35 species found in the Americas, 11 in Africa, and 6 in Asia, totalling approximately 200 arthropod species that feed on cassava [9].

Whiteflies are one of the main pests of cassava worldwide, as they cause considerable damage by piercing leaves and sucking sap, resulting in chlorosis, deformation, defoliation, and sooty mould growth. The overall outcome of these effects is plant stunting and a reduction in storage root yields [10,11,12,13]. The most impactful indirect damage is the transmission of numerous plant diseases such as cassava mosaic disease (CMD) caused by cassava mosaic geminiviruses (CMGs), one of the most devastating diseases of crops and one of the main constraints to cassava cultivation, and cassava brown streak disease (CBSD), a particularly devastating disease because it negatively affects the tuberous roots of cassava, both quantitatively and qualitatively [10,11,12,13]. CMD and CBSD are the most important cassava diseases in terms of economics, causing annual yield losses of over USD 1 billion [13,14,15]. There are few chemical control options for whitefly species, and there is little knowledge of their natural enemies. Hence, most of the limited research into whitefly management has been exploratory and focused on potential and environmentally friendly methods, such as biological control, crop management, and host–plant resistance [16,17]. In the Neotropics, *Aleurotrachelus socialis* Bondar (Hemiptera: Aleyrodidae) and *Aleurothrixus aepim* (Goeldi, 1886) (Hemiptera: Aleyrodidae) are the most devastating cassava pests [18]; in northeastern Brazil, the most common whitefly species causing severe damage to cassava is *A. aepim* [19].

Plants generally have wide-reaching and highly dynamic biochemical defence mechanisms against herbivory, mediated by both direct and indirect pathways. Defensive compounds are produced either constitutively or in response to plant damage, and they affect herbivore feeding, growth, and survival [20,21,22]. Plant defence mechanisms involve the release of volatile organic compounds (VOCs), which play essential roles in communication between plants and other organisms [23,24,25,26]. VOCs are linked to defence against herbivores and pathogens. Additionally, they are involved in the attraction of pollinators, seed dispersers, and beneficial microorganisms, as well as in communication signals between plants [26,27,28,29]. Fundamental processes, such as the effect of volatile compound blends on the behaviour of specific insects, make plants attractive or repellent to herbivorous insects [30,31]. Cassava plants also emit VOCs and secrete extrafloral nectar; these substances may be essential for developing biological control strategies against pests [32,33]. For example, *Bemisia tabaci* (Genn.) (Hemiptera: Aleyrodidae) can carry plant-derived detoxification genes, which is a new evolutionary scenario whereby herbivores harness the genetic toolkit of the host plants to develop resistance to plant defences; hence, this pest can be investigated and exploited for crop protection [34]. Several cassava cultivars with varying levels of whitefly resistance have been identified. Cassava varieties with different concentrations of cyanogenic glycosides are less preferred by *B. tabaci* whiteflies [35]. Nymph mortality was found to be higher in the resistant cassava clone, M Ecuador 72, than in other clones. When *A. socialis* and *Bemisia tuberculata* (Bondar, 1923) (Hemiptera: Aleyrodidae) whiteflies fed on this genotype, they exhibited lower oviposition rates, extended developmental periods, reduced size, and higher mortality compared to those feeding on other genotypes. Breeding programs have used M Ecuador 72 as a source of resistance; moreover, many resistant hybrids have been generated using this clone as the female parent [36,37,38]. In this study, we investigated the role of VOCs in the resistance of M Ecuador 72 to *A. aepim* whiteflies, the constitutive VOCs released by this clone and a susceptible cassava genotype, and the influence of VOC extracts and an individual compound, (*E*)-β-ocimene, on whitefly behaviour.

## 2. Materials and Methods

### 2.1. Plants

Cassava genotypes BRS Jari and M Ecuador 72 were collected from Embrapa Cassava and Fruticulture (12°40′48.2″ S, 39°05′21.1″ W), Cruz das Almas City, Bahia State, Brazil, in August 2018 during the dry season. The greenhouse (Van der Hoeven, Van der Hoeven Estufas Agricolas Ltda, Artur Nogueira, SP, Brazil) growth conditions were a temperature of 25 ± 2 °C with 70% relative humidity and a 14 h/10 h (day/night) photoperiod. All plant materials were vegetatively propagated (stem cuts), grown in 0.5 L plastic pots containing a commercial planting mix (Bioplant^®^, Bioplant Misturadora Agrícola Ltda, Nova Ponte, MG, Brazil) substrate, and then manually watered every 1 or 2 days when necessary until they reached an age of 40–45 days, the stage of VOC extraction, and had 6–7 leaves.

### 2.2. Insects

Adult whiteflies (*A. aepim*) undifferentiated by sex were collected from a cassava plantation located in the municipality of Rio Largo, State of Alagoas, Brazil, at the geographical coordinates of 9°31′50.1″ S 35°48′1.3″ W. After collection from the field, the insects were sent to the laboratory, where they were identified, for behavioural bioassays. They were kept in small glass tubes in an air-conditioned room at 25 ± 2 °C and 70% ± 10% relative humidity (RH). The species was identified by PCR amplification of a partial mtDNA COI gene and sequence comparison using BLASTN searches against a nonredundant DNA database in GenBank (MT541892).

### 2.3. Dynamic Headspace Collection

Cassava plants were maintained in cages without whitefly infestation. VOCs were collected using sterile plastic cooking bags (Qualitá cooking bags composed of polyester [poly(ethylene terephthalate) or PET], 27 × 41 cm, max 200 °C [RMBPACK Machines and Packaging Ltd., Colombo, PR, Brazil]). After cooking, identical precleaned polyester bags were opened and baked in an oven (100 °C) for 2 h. The bags were inflated with clean air and subsequently deflated (thrice) to remove any residual contamination, and the VOCs were collected using a push–pull system. Six cassava plants in six identical precleaned plastic bags were used for each extraction. The VOCs were trapped on a Porapak Q (50/80 mesh, 0.05 g, Supelco Inc., Bellefonte, PA 16823, USA). The absorbent was placed in a glass tube and inserted at the top of the bag. Air was filtered through activated charcoal and then pumped into the plastic bag at a flow rate of 600 mL/min per plant before being collected at a flow rate of 400 mL/min. After the collection was complete, the trapped VOCs were desorbed with 500 µL of double-distilled hexane (HPLC grade), and the samples were stored at −20 °C until analysis and bioassay. The samples were collected in the laboratory for 60 h.

### 2.4. Gas Chromatography–Mass Spectrometry (GC-MS) Analysis

The volatile extracts were analysed by coupled GC–MS (mass spectrometry) using a GCMSQP 2010 Ultra instrument (Shimadzu, Kyoto, Japan), fitted with a gas chromatograph (column specifications: 30 m × 0.25 mm i.d., film thickness 0.25 μm, J & W Scientific, Santa Clara, CA, USA). Helium was used as the carrier gas at a flow rate of 1 mL/min. The samples were injected into a splitless injector at the temperature of 200 °C with the injections of 1 µL being performed in splitless mode. The oven temperature was programmed to start at 50 °C for 5 min before rising to 250 °C at a rate of 5 °C/min, with a final hold time of 5 min and a 50 min total run time. Electron impact ionisation was conducted at 70 eV, with the temperature of the ion source and detector set at 200 °C. The interface temperature was 250 °C, whereas the recorded fragmentation values were scanned from 35 to 300 *m*/*z*. Plant VOCs were identified by analysing their mass spectra and comparing them with those in the NIST 08 library and then those of authentic standards (ocimene mix of isomers, *trans*-caryophyllene, and farnesol; Sigma-Aldrich, St. Louis, MO, USA). The compounds were quantified based on the peak area relative to the internal standard (nonacosane) provided by Sigma-Aldrich. Each treatment employed six replicates.

### 2.5. Olfactometer Assays

#### 2.5.1. Y-Tube Olfactometer

A Y-tube olfactometer comprising a glass tube with 1.5 cm internal diameter and Y-section arms with lengths of 7.5 cm was used in the horizontal position. The angle between the two Y branches was 60°, whereas each arm of the ‘Y’ was 15 cm from the junction. Purified air was drawn through each unit to the central arm at a flow rate of 50 mL/min.

#### 2.5.2. Odour Treatments

The olfactory response of the whiteflies was investigated using VOC extracts in hexane (genotypes: BRS Jari and M Ecuador 72) and at concentrations of 0.3, 3, 300, and 3000 ng/µL (*E*)-β-ocimene, a compound found at higher levels among VOCs of the resistant genotype. This procedure was similar to that described in previous reports [39,40]. Before performing the behavioural bioassays, the field-collected whiteflies were left to settle for 2 h. All doses of (*E*)-β-ocimene were prepared by diluting them in appropriate amounts of double-distilled hexane (HPLC grade). Aliquots (10 μL) of each solution were used for testing. Each sample to be tested was added to a filter paper (1.5 × 1 cm) and then placed in one of the arms, and an aliquot of double-distilled HPLC-grade hexane (10 μL) was added to a similar filter paper and placed in the other arm. Therefore, one arm was used to release the treatment for each experiment, and the other was used as a solvent control. The solvent was evaporated from the filter paper for 30 s before commencing the experiments.

After the airflow began, one adult whitefly was introduced into the olfactometer for 30 s. The choice was made when the whitefly crossed half the arm’s length of the odour source or control. A ‘no-choice’ decision was made if the whitefly did not move for 5 min. Each piece of filter paper was used only once and replaced with a new piece for each insect. Every insect was used only once in the experiments. The sides on which the treatment and control groups were presented were swapped to avoid positional bias. The olfactometer was replaced after three whiteflies were tested. Each treatment group consisted of at least 30 adult whiteflies.

### 2.6. Chemometric Analysis

The data acquired for analysis were processed using GCMSsolution workstation software, and the chromatograms were aligned. The metabolic profile data were organised in a spreadsheet (Microsoft Excel 2021, Version 365) in which the identified compounds were arranged in columns and sample names in rows, forming a data matrix. The peak areas of the VOCs obtained from the chromatograms were then normalised to the sum treated on the cube root transformation scale, which transformed the response variable from y to y^1/3^. This provided a better normal distribution of the data. The data were also scaled according to the Pareto scale using MetaboAnalyst 4.0. Multivariate chemometric analyses, including partial least squares–discriminant analysis (PLS-DA) and orthogonal projections to latent structure–discriminant analysis (OPLS-DA), were performed, and heat maps and hierarchical cluster analysis (HCA) graphs were constructed. The procedure was repeated several times to calculate the performance and confidence intervals of each model. The analyses were performed using MetaboAnalyst 5.0, a web-based tool, according to the protocols provided in the literature [41].

### 2.7. Statistical Analysis

The cassava volatiles from both genotypes were analysed using the Kolmogorov–Smirnov and Shapiro–Wilk normality tests, and since they were not normalised, they were subjected to the Wilcoxon–Mann–Whitney test (*p* < 0.05). The behavioural bioassay responses were analysed using the chi-square test, considering significance levels of *p* < 0.05 or *p* < 0.01. Analyses were performed using the GENES software [42].

## 3. Results

### 3.1. Different Genotypes Constitutively Express Different VOC Blends

**Figure 1:**
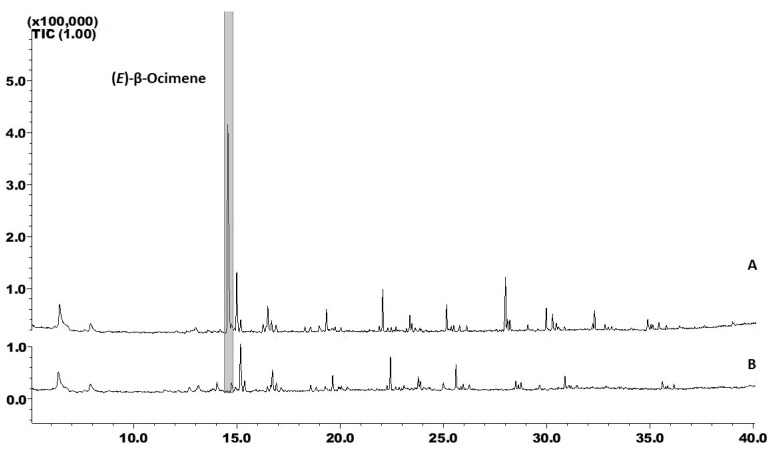
Gas chromatography–mass spectrometry analyses of volatile extracts collected from cassava plants (A) M Ecuador 72 (Resistant) and (B) BRS Jari (Susceptible). Volatiles were collected for 60 h using Porapak Q as an adsorbent.

Analyses of the VOC emission profiles of M Ecuador 72 (resistant) and BRS Jari (susceptible) (Figure 1) revealed a variety of compounds from different chemical classes and functions, such as terpenes, alkanes, branched alkanes, alkenes, ketones, aldehydes, and alcohols. Twenty-five volatile compounds were detected using GC-MS, of which twelve were exclusive to M Ecuador 72 and five to BRS Jari (Table 1).

M Ecuador 72 produced 22 compounds, whereas BRS Jari produced 15 compounds. The common compounds were *n*-alkanes (C15–C16), (*E*)-β-ocimene (4), linalool (6), (*E*)-caryophyllene (17), and methyl salicylate (10). Some compounds, namely, 4-octen-3-one (2), 3-ethylacetophenone (12), dodec-1-ene (11), tetradec-1-ene (16), farnesol (24), and (*Z*)-β-ocimene (3), were exclusively found in M Ecuador 72 (Table 1) and may have been responsible for the better resistance of this genotype to whiteflies. Compared with BRS Jari, M Ecuador 72 produced significantly more (*E*)-β-ocimene (4). After 60 h of collection, this compound had a concentration of 250.20 ng per plant in M Ecuador 72 and 105.43 ng per plant in BRS Jari.

### 3.2. Behavioural Responses of Whiteflies to Volatiles Emitted by Two Cassava Genotypes

**Figure 2:**
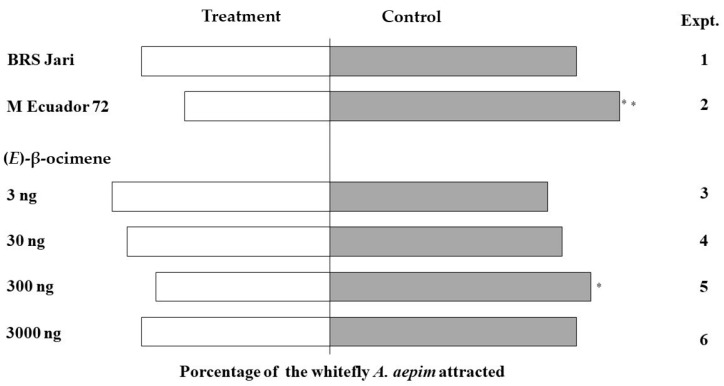
Behavioural response of *Aleurothrixus aepim* whiteflies in a Y-olfactometer to VOCs collected for a period of 60 h and to (*E*)-β-ocimene. Experiments 1 and 2 employed BRS Jari and M Ecuador 72 extracts. For Experiments 3–6, doses of 3, 30, 300, and 3000 ng of (*E*)-β-ocimene (4) standard were used. Data were analysed using GENES software (* *p* < 0.05; ** *p* < 0.01).

The results obtained in the behavioural bioassays showed that the whiteflies preferred the control odour (hexane) to the extract from the resistant genotype, M Ecuador 72 (Figure 2). The VOCs from M Ecuador 72 exhibited significantly more repellent activity (χ^2^ = 11.00; *p* < 0.01, N = 30) than those from BRS Jari (χ^2^ = 3.24; *p* > 0.05, N = 30) against the same control (hexane). We also tested the repellent activity of a series of (*E*)-β-ocimene (4), the main VOC in the resistant genotype. The flies showed the greatest preference for the control arm (χ^2^ = 4; *p* = 0.0455) in the bioassay with 300 ng of the standard compound. The Y-tube olfactometer results also demonstrated that the resistant genotype exhibited repellent activity against *A. aepim* whiteflies (*p* < 0.000911). Considering its high emission rates, we also tested pure (*E*)-β-ocimene (4) in the same bioassay. Our results showed that (*E*)-β-ocimene (4) was avoided by the whiteflies tested, suggesting that it may be one of the main active components responsible for the repellent activity of M Ecuador 72.

### 3.3. Comparative Analysis of the VOC Profiles of M Ecuador 72 and BRS Jari

**Figure 3:**
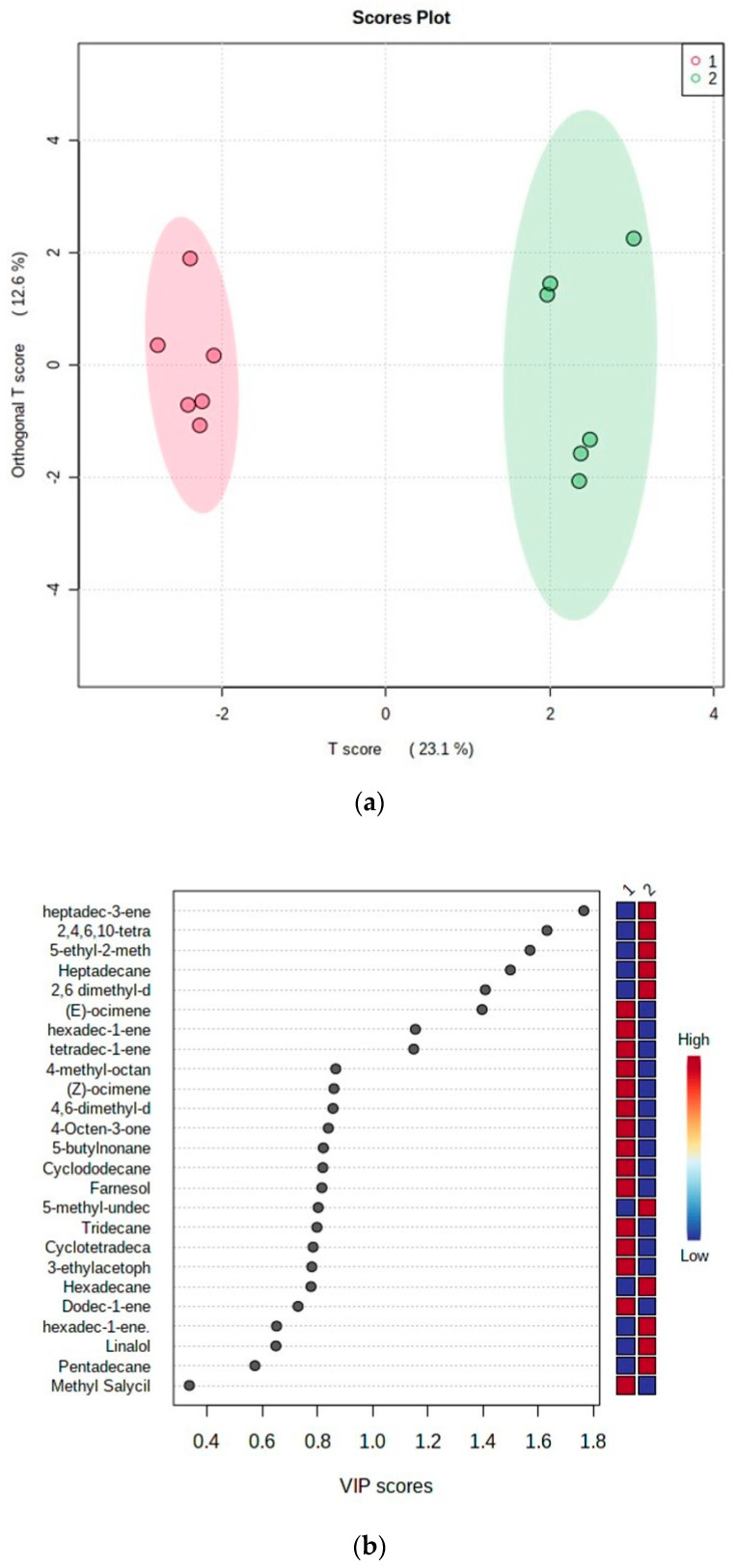

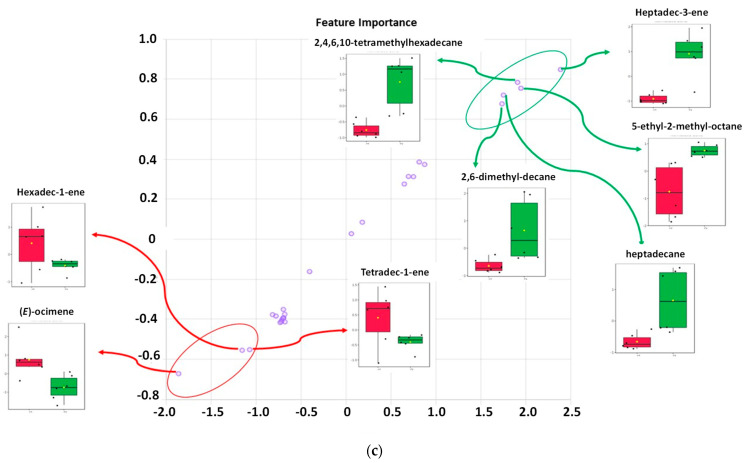
Graphs obtained from a comparative analysis of the volatiles collected from non-infested plants of the M Ecuador 72 (resistant to whiteflies) (red) and BRS Jari (susceptible to whiteflies) (green) genotypes during a period of 60 h: (**a**) OPLS-DA score; (**b**) S-Plot; (**c**) VIP score.

The OPLS-DA score; S-Plot; VIP score (Figure 3) showed differences between the profiles of the VOCs emitted by the two genotypes over a period of 60 h of analysis; there was a clear distinction between the samples from both genotypes in the two groups. Samples of M Ecuador 72 exhibited negative values (red) for component 2. Samples of BRS Jari exhibited positive values (green) for the same component. OPLS-DA models were constructed to identify the VOCs responsible for the distinction between the two genotypes. The OPLS-DA score graph (Figure 3a) explained 35.9% of the total variance and showed that the genotypes behaved differently with regard to the biosynthesis and emission of metabolites in non-infested plants. The values of the quality parameters for the model were satisfactory (R2Y = 0.82 and Q2 = 0.607), suggesting a statistically significant difference between the metabolic profiles of the analysed samples. The S-plot dispersion graph (Figure 3b) also showed the variables responsible for the group separation observed in the score graph. The compounds that characterised the resistant genotype (M Ecuador 72) were located on the negative axis. On the positive axis were metabolites related to the susceptible genotype (BRS Jari). The VIP score (Figure 3c) corroborated the results obtained from the OPLS-DA S-plot, which presented the VOCs with repellent activity already reported in the literature [43,44,45,46]. The monoterpene (*E*)-β-ocimene (4) and the hydrocarbons tetradec-1-ene (16) and hexadec-1-ene (19) were the most abundant in the samples of the resistant genotype and were highlighted as biomarker candidates for its resistance.

## 4. Discussion

Plants have been reported to produce and release VOCs into the environment in response to herbivory [47]; such emissions can help ward off herbivores by attracting their natural enemies [31,48,49]. Plants that emit VOCs can protect neighbouring crops belonging to the same or different species by communicating through VOC receptors [50,51,52].

Herein, we described variations in the blend constitution and individual concentrations of VOCs released by two cassava genotypes. The genotype M Ecuador 72 has been described as highly resistant to *B. tabaci*, *A. socialis*, and *B. tuberculata* [36,53,54]. Such resistance has been explained based on insect biology and behavioural parameters, such as the higher nymphal development time, lower survival rate, and smaller size of *B. tuberculata* [37], as well as the lower oviposition rates, reduced size and fecundity, and higher mortality of *A. socialis*, compared to those of other insects [36,53]. The higher mortality and extended development time of the immature phase of *A. aepim* have previously been observed in M Ecuador 72 [55]. However, the role of the metabolites involved in the resistance of this genotype to whiteflies has received little attention, and studies on the effects of these metabolites on insect behaviour and biology are lacking. Cassava is a commercial crop that exhibits resistance to whiteflies [53,56]; therefore, understanding the mechanisms and sources of such resistance is essential for whitefly management. The biology of whiteflies in their interaction with M Ecuador 72 suggests the involvement of semiochemicals. However, the ecological role of VOCs in cassava has rarely been explored. In this study, M Ecuador 72 was found to release a higher number of compounds and at a higher intensity than the BRS Jari genotype (Figure 1), as observed in different maize varieties. These genotypes also demonstrated intraspecific variations in odour quality and quantity [57]. The VOC blend from M Ecuador 72 plants showed a higher number of compounds with reported biological activity in other systems, such as linalool, farnesol, dodec-1-ene, 1-tetradec-1-ene, hexadec-1-ene, pentadecane, and 3-ethylacetophenone, in addition to the monoterpene (*E*)-β-ocimene. Varietal and genotypic differences in herbivore-induced plant volatiles (HIPVs) have been widely reported [58,59,60,61]. Constitutive VOCs play an essential role in plant defence by attracting natural enemies of pests as part of the systemic defence response. Herein, we have highlighted the constitutive variation in the VOC profiles of M Ecuador 72 and a susceptible genotype, BRS Jari. The latter emits volatile compounds such as phytane, heneicosane, and heptadecane, similar to healthy *Rosa chinensis* Jacq. plants, which easily contract powdery mildew [62]. Furthermore, the heneicosane and other long-chain alkanes found in this cultivar are attractive to *Callosobruchus maculatus* (Fabricius) (Coleoptera: Bruchidae), a critical seed pest of *Lathyrus sativus* L. [63], which can be connected to the susceptibility of this cultivar to whiteflies. Plant VOCs are essential for parasitoids and predators to locate hosts [31]. For example, dodecene attracts the parasitoid *Chouioia cunea* Yang to control the pupae of the fall webworm, *Hyphantria cunea* (Drury) [64], whereas tetradecane and hexadecene have been found in the faecal odours of the larvae of the stored grain pest, *Tribolium confusum* du Val (Insecta: Coleoptera: Tenebrionidae), which attracts the parasitoid *Holepyris sylvanidis* (Hymenoptera: Bethylidae) [65]. *Caragana ordosica* (Fabaceae) emits pentadecane and other compounds that repel the insect *Chlorophorus caragana* (Coleoptera: Cerambycidae) [66]. 3-Ethylacetophenone is a VOC in aphid-infested plants that attracts the adult multi-coloured Asian lady beetle, *Harmonia axyridis* (Pallas, 1773) (Coleoptera: Coccinellidae) [67]. The sesquiterpene, farnesol, emitted by *Lantana* plants, acts as a synomone (produced and released by one species that benefits the emitter and receiver) to attract an omnivorous predator, *Campylomma chinensis* Schuh (Hemiptera: Miridae), which has considerable potential to suppress whiteflies [68]. Farnesol attracts natural enemies of pests and has the potential to act as a repellent against *Myzus persicae* (Sulzer) (Hemiptera: Aphididae) [69,70,71]. Further studies are necessary to investigate the roles of farnesol and linalool in cassava plants. One strategy for the Integrated Pest Management (IPM) of whiteflies is to maintain the level of infestation below a certain economic threshold [72]. Therefore, the identification of genotypes with repellent activity could be a component of a more efficient IPM strategy.

In this study, we examined the activity of a monoterpene, given that this compound was emitted in higher concentrations by the resistant genotype. The repellent properties of M Ecuador 72 against *A. aepim* may have been a result of the individual actions of one or more VOCs. (*E*)-β-ocimene acted as a repellent when tested individually. It is a standard VOC released from the leaves and flowers of many plants [73] and can play several biological roles by potentially mediating defensive responses to herbivory. As an indirect defence mechanism, (*E*)-β-ocimene has been described as a chemical cue for natural enemies of phytophagous insects, including parasitoids and predators [72]. Pre-exposure to the natural compound, *Z*-jasmone, induces the emission of (*E*)-β-ocimene in Chinese Cabbage (*Brassica pekinensis* Skeels) [43]. Bioassays have confirmed the attraction of the parasitoid *Aphidius ervi* Haliday, 1834 to the (*E*)-β-ocimene standard [72]. This compound also exhibits critical biological activity in plant–plant interactions. Exposure of *Arabidopsis thaliana* (L.) Heynh plants to a blend of ocimene volatiles triggers defence responses via signalling hormone pathways, such as the methyl jasmonate pathway [74]. When *B. pekinensis* was treated with (*E*)-β-ocimene, both direct and indirect defence responses were induced [43]. In that study, the growth and reproduction of the aphid *M. persicae* were directly and significantly affected by the accumulation of defence-related metabolites. Moreover, the parasitoid *Aphidius gifuensis* Ashmead, 1906 showed a preference for treated plants and, in terms of olfaction and landings, performed more successfully on treated plants than on healthy plants [43]. Ocimene is a monoterpene found in nature, and (*E*)-β-ocimene is known to exhibit anticonvulsant, antifungal, antitumor, and pest-resistant properties [75]. Medical cannabis has a high ocimene content. [76]. *Brassica pekinensis* plants treated with ocimene negatively influence the feeding behaviour of *M. persicae*. In addition, ocimene increases attraction from the parasitoid *Aphidius gifuensis* (Hymenoptera: Braconidae) [43,45]. The results of behavioural bioassays using tea plants (*Camellia sinensis* cv. Shuchazao) demonstrated that (*E*)-β-ocimene strongly repelled mated *Ectropis obliqua* (Prout, 1915) (Lepidoptera: Geometridae) females [46]. Ocimene is an acyclic compound that, in addition to its biologically attributed functions in plant protection, affects floral visitors and pest resistance by mediating defensive responses to herbivory. (*E*)-β-ocimene is a crucial plant VOC with multiple functions in plants, depending on the organ and time of emission, and plays relevant roles in establishing tri-trophic interactions.

The results of the Y-tube olfactometer assay in this study revealed that *A. aepim* whiteflies showed non-preferential behaviour toward the extracts of the resistant genotype but not those of the control. Similar results were obtained previously; several celery varieties displayed strong repellence against *B. tabaci,* and (*E*)-β-ocimene was identified as one of their main active compounds [41]. Most studies to date have focused on the attractive properties of HIPVs rather than on the repellence activity of plant VOCs [77]. In the push–pull strategy, the pest is repelled or deterred (pushed). Herein, we have demonstrated the non-preferential response to the resistant cassava clones and suggested exploring the potential of the constitutive VOCs based on genetic variability. Plant breeding may impair beneficial interactions involved in VOC-mediated protection. Teosintes (wild maize species) show higher resistance to many pests than cultivated maize [66,67]. Quantitative and qualitative evaluation of 31 inbred maize lines indicated high levels of odour variability [78]. Additionally, 25 landraces, 30 hybrids, and 22 inbred maize lines were screened for HIPV emissions after oviposition and activity in the egg parasitoid, *Cotesia sesamiae* (Cameron) (Hymenoptera: Braconidae) [78]. The authors found high variability, especially among landraces, and suggested that the introgression of such traits into commercial varieties occurred because of their high protection potential. 

The difference between the constitutive VOCs from the cassava genotypes selected for this study suggests that further investigation of the variability in odours from cassava genotypes and the verification of VOC emissions under field conditions are necessary. The judicious use of insecticide is required to manage pests and vectors, and we have shown that constitutive VOCs from cassava plants could serve as a component of IPM for whiteflies, specifically *A. aepim*.

## Figures and Tables

**Table 1:** Compounds from the cassava genotypes M Ecuador 72 (resistant to whiteflies) and BRS Jari (susceptible to whiteflies). Each compound was quantified using nonacosane internal standards (mean ± SE in ng/µL) with the Wilcoxon–Mann–Whitney test, *p* < 0.05.

No	Compounds	RI	M Ecuador 72 (ng/µL)	BRS Jari (ng/µL)
1	4-Methyl-octane	857	26.02 ± 11.64	-
2	4-Octen-3-one	958	19.47 ± 8.73	-
3	(*Z*)-β-ocimene	1044	18.33 ± 8.20	-
4	(*E*)-β-ocimene	1046	250.20 ± 32.29 ^a^	105.43 ± 16.98 ^b^
5	5-Ethyl-2-methyl-octane	1052	27.43 ± 12.78 ^a^	58.80 ± 11.76 ^a^
6	Linalool	1094	21.04 ± 9.41 ^a^	27.26 ± 8.71 ^a^
7	2,6 Dimethyl-decane	1129	-	26.83 ± 12.89
8	5-Butyl-nonane	1152	19.28 ± 8.62	-
9	5-Methyl-undecane	1153	18.61 ± 8.32 ^a^	22.48 ± 10.55 ^a^
10	Methyl salicylate	1188	39.35 ± 8.32 ^a^	21.35 ± 9.56 ^a^
11	Dodec-1-ene	1193	19.29 ± 8.62	-
12	3-Ethylacetophenone	1281	22.42 ± 10.03	-
13	Tridecane	1300	18.59 ± 8.31	-
14	4,6-Dimethyl-dodecane	1321	26.84 ± 12.00	-
15	Cyclododecane	1328	18.60 ± 8.31	-
16	Tetradec-1-ene	1393	24.75 ± 7.82	-
17	(*E*)-β-caryophyllene	1417	38.16 ± 0.58 ^a^	28.94 ± 9.71 ^a^
18	Pentadecane	1500	33.28 ± 10.96 ^a^	33.79 ± 13.01 ^a^
19	Hexadec-1-ene	1586	18.76 ± 8.39 ^a^	19.86 ± 8.90 ^a^
20	Hexadecane	1600	20.20 ± 9.04 ^a^	29.97 ± 15.92 ^a^
21	Cyclotetradecane	1671	18.88 ± 8.44	-
22	Heptadec-3-ene	1676	-	42.25 ± 9.54
23	Heptadecane	1700	-	25.21 ± 12.44
24	Farnesol	1719	18.96 ± 8.47	-
24	Octadecane	1800	26.45 ± 8.39 ^a^	20.25 ± 9.07 ^a^
25	Phytane	1811	-	28.12 ± 9.31
26	Heneicosane	2100	-	25.68 ^±^ 8.15

The amounts were calculated based on an internal standard (nonacosane). Means followed by different letters are significantly different according to the Wilcoxon–Mann–Whitney test (*p* < 0.05).

## Data Availability

The data presented in this study are available on request from the corresponding author.
